# Selection of Reference Genes for Gene Expression Studies in Siberian Apricot (*Prunus sibirica* L.) Germplasm Using Quantitative Real-Time PCR

**DOI:** 10.1371/journal.pone.0103900

**Published:** 2014-08-08

**Authors:** Jun Niu, Baoqing Zhu, Jian Cai, Peixue Li, Libing Wang, Huitang Dai, Lin Qiu, Haiyan Yu, Denglong Ha, Haiyan Zhao, Zhixiang Zhang, Shanzhi Lin

**Affiliations:** 1 College of Biological Sciences and Biotechnology, College of Nature Conservation, National Engineering Laboratory for Tree Breeding, Key Laboratory of Genetics and Breeding in Forest Trees and Ornamental Plants, Ministry of Education, Beijing Forestry University, Beijing, China; 2 Jigongshan National Nature Reserve, Xingyang, China; 3 Research Institute of Forestry, Chinese Academy of Forestry, Beijing, China; ISA, Portugal

## Abstract

Quantitative real time reverse transcription polymerase chain reaction has been applied in a vast range of studies of gene expression analysis. However, real-time PCR data must be normalized with one or more reference genes. In this study, eleven putative consistently expressed genes (*ACT*, *TUA*, *TUB*, *CYP*, *DNAj*, *ELFA*, *F-box27*, *RPL12*, *GAPDH*, *UBC* and *UBQ*) in nine Siberian Apricot Germplasms (including much variability) were evaluated for their potential as references for the normalization of gene expression by NormFinder and geNorm programs. From our studies, *ACT*, *UBC*, *CYP*, *UBQ* and *RPL12* as suitable for normalization were identified by geNorm, while *UBC* and *CYP* as the best pair by NormFinder. Moreover, *UBC* was selected as the most stably expressed gene by both algorithms in different Siberian Apricot seed samples. We also detected that a set of three genes (*ACT*, *CYP* and *UBC*) by geNorm as control for normalization could lead to accurate results. Furthermore, the expression levels of oleosin gene were analyzed to validate the suitability of the selected reference genes. These obtained experimental results could make an important contribution to normalize real-time PCR data for gene expression analysis in Siberian Apricot Germplasm.

## Introduction

Quantitative real time reverse transcription polymerase chain reaction (RT-qPCR) has replaced the classical reverse transcription-polymerase chain reaction with quantitative accuracy, high sensitivity and high-throughput characteristics, thus becoming the most common method for detection and quantification of mRNA transcription levels of target genes [1]. However, obtaining accurate and reliable quantitative gene expression results is difficult. This is due to experimental variation, such as differences in amount and quality of starting material, quantity and quality of RNA, and enzymatic efficiencies during reverse transcription [2]. Because reference genes undergo the same preparation steps as the target gene, by selecting one or more reference genes used for normalization in real-time PCR analysis this issue can be avoided. These reference genes under selection could potentially stabilize the experimental variability that typically occurs as a result of the various steps of the experimental procedure [3]. Certainly, the expression levels of reference genes should remain constant between the cells of different varieties, tissues, lifecycle phases and experimental conditions, otherwise, it can lead to erroneous results in quantification of the interesting gene [4].

The housekeeping genes, such as 18S rRNA, actins (*ACT*), cyclophilin (*CYP*), tubulin (*TUA* and *TUB*) and glyceral-dehyde-3-phosphate dehydrogenase (*GAPDH*), were typically used as reference genes for normalization of mRNA expression owing to their stable expression [5], [6]. However, studies have shown that the expressions of those commonly used reference genes will not display a stable expression under some experimental conditions, for example, a reference gene may be stably expressed in one organism but unsuitable for normalization of gene expression in another [7]. Therefore choosing suitable reference genes for normalization relative to certain experimental materials and conditions is crucial. In recent years, many statistical algorithms, such as NormFinder [8], BestKeeper [9] and geNORM [10], have been extensively applied to evaluate ideal reference genes for normalizing real-time PCR data according to the specific tissue type and experimental conditions [11], [12].

Siberian Apricot (*Prunus sibirica* L.), belonging to the Rosaceae, is a deciduous shrub native to temperate, continental, mountainous climates including regions of northern and northeastern China, eastern and southeastern portions of Mongolia, Eastern Siberia and the Maritime Territory of Russia [13]. It grows in temperate climates and thrives with abundant sunlight, low temperatures, strong winds, low rainfall and poor soil. In China, Siberian Apricot is one of the most economically and ecologically important tree species owing to its plentiful resource, fast growth, annual seed production more than 192,500 tons, high oil content in seed kernel, and a wide range of uses [14]. Although the 26S ribosomal RNA gene was used as the sole reference gene for examining carotenoid biosynthesis in apricot during a recent expression survey [15], the results have not been entirely validated.

The aim of this study was to select and evaluate the stability of 11 candidate reference genes using different Siberian Apricot Germplasm seeds in China by statistical software (NormFinder and geNorm). The optimal reference genes might be applied in further studies on the Siberian Apricot gene expression for selecting high-quality seeds at the molecular and genetic level. Furthermore, the expression levels of oleosin gene were assessed using different reference genes, to validate the selection of candidate reference genes.

## Materials and Methods

### Ethics Statement

All materials are in a natural state, and specific permission was not required for these locations/activities. No protected plant species was sampled for this research.

### Tissue collection

Based on an overall investigation of Siberian Apricot in China, 9 superior trees (Donglin, Lanxian, Fuxing, Zhidan, Keqi, Tianjing, Yanji, Zhenyuan and Minghe) have been selected from each tree population (Figure S1). Selections were based on phenotypic assessment of agronomic morphological characteristics of economic interest, including seed yields per plant, kernel rate of seed, number of fruit clusters per plant, number of fruits per cluster, disease resistance, and so forth. A global positioning system (Garmin GPSMAP76) was used to mark the position of the collected accessions. Although the experimenters did not study the effect of each single factor on candidate reference genes stability, an overall assessment indicated a fair degree of variability among the trees analyzed, at least on climatic and environmental factors (Table 1). Table 1 shows the specific location, meteorological data and edaphic condition of the Siberian Apricot Germplasms. Fully ripened fruits of Siberian Apricot were harvested at maturity stage in the 2011 harvest season. The sarcocarp of all Siberian Apricot seeds was removed, and they were immediately immersed in liquid nitrogen and stored at −80°C until RNA extraction.

**Table 1:** The data of material location, meteorological data and edaphic condition.

Germplasm*	longitude/°	latitude/°	Altitude/m	Annual averaged temperature/°C	mean temperature (Jan)/°C	mean temperature (July)/°C	Annual rainfall/mm	Non-frost/d	Sunshine duration/h	Soil
Donglin	131°08.086′	44°08.200′	382	4.9	−14.6	21.5	530	151	2322	Dark Brow Soil
Keqi	117°32.361′	43°24.030′	1303	2.5	−25.0	20.0	380	95	2825	Cinnamon Soil
Yanji	129°23.102′	42°50.209′	326	5.7	−14.4	21.3	533	140	2317	Dark Brow Soil
Fuxing	121°22.121′	42°09.387′	289	7.0	−11.6	24.2	494	150	2623	Cinnamon Soil
Tianjing	117°23.952′	40°09.231′	194	11.3	−5.5	26.0	518	195	2778	Cinnamon Soil
Lanxian	111°29.357′	38°08.920′	1655	6.8	−9.7	21.0	500	126	2861	Raw Soil
Zhidan	108°45.741′	36°49.025′	1366	7.8	−7.9	21.5	525	142	2313	Loessal Soil
Minghe	102°53.545′	36°11.090′	2043	9.0	−6.4	19.8	292	198	2458	Chestnut Soil
Zhenyuan	107°13.601′	35°35.159′	1280	9.9	−5.0	20.8	515	155	2350	Red Clay

*All of the trees are full bearing period.

### RNA extraction and cDNA preparation

Total RNA was isolated from tissue samples using the RNeasy Fibrous Tissue Mini Kit (QIAGEN) according to the manufacturer's recommendations. The RNase-free DNase kit (Promega) was subsequently used for eradication of DNA contamination in total RNA preparations. Extracted RNA was qualified and quantified using a Nanodrop ND-1000 Spectrophotometer (Nanodrop Technologies, Wilmington, DE, USA) and all the samples showed a 260/280 nm ratio from 1.9 to 2.1. Equal amounts of total RNA (1.5 µg) in all samples were reverse transcribed respectively using the Reverse transcription System (Promega) in a 20 µl reaction using oligo-dT primers, according to the manufacturer's instructions, and then the cDNA was diluted 1∶5 with nuclease-free water before being used as templates in the qPCR process.

### Selection of Siberian Apricot sequences and PCR primer design

To identify Siberian Apricot homologs of internal genes, such as *ACT*, *TUA*, *TUB*, *CYP*, chaperone protein danJ (*DNAj*), elongation factor 1-alpha (*ELFA*) and polyubiquitin (*UBQ*), we queried expressed sequence tag (EST) databases with *Prunus amygdalus*, *P. maritima* and *P. avium* nucleotide sequence using nucleotide blast, then homologous comparison gene sequences were performed to confirm the function of selected apricot ESTs. The rest of apricot reference genes could be found in NCBI nucleic acid database. The amplification primers for real-time PCR were designed using the Primer Premier 5 software with melting temperatures between 58 to 62°C, and the absence of secondary structures was verified by the UNAFold program (http://eu.idtdna.com/UNAFold) according to D'haene et al [16]. Most of the primer pairs were targeted to different exons or spanned 2 exons in order to avoid false positives during amplification due to potential residual genomic DNA in the templates. Specificity of each sequence primer pair was verified by conventional RT-PCR under the same conditions as described below for real-time RT-PCR, after that, products were confirmed by gel electrophoresis on 1.5% agarose gel and visualized after staining with ethidium bromide.

### Real-time RT-PCR and data analyses

Real-time amplification reactions using SYBR Green were performed with the ABI 7500 Fast Real-Time PCR System (Applied Biosystems). Reactions were prepared in a 20 µl volume containing: 4 µl of template, 0.8 µl of each amplification primer (0.4 µM), 0.4 µl of ROX Reference DyeII, 10 µl of 2×SYBR premix Ex Taq™II (TaKaRa Biotechnology) and 6 µl of dH_2_O. Each PCR reaction was performed in triplicate and template-free negative controls were performed simultaneously.. Amplifications were performed starting with an initial step of 95°C for 30 s, followed by 40 cycles of denaturation at 95°C for 3 s and primer annealing at 60°C for 30 s. Melting curve analysis ranging from 60°C to 95°C with temperatures increasing in increments of 0.2°C every 10 s was performed for all PCR products using ABI Prism Dissociation Curve Analysis Software to confirm the occurrence of specific amplification peaks. The PCR efficiency (E) was estimated from the data obtained from the exponential phase of each individual amplification plot and the equation (E = 10^slope^) [17]. Negative controls consisting of nuclease-free water instead of template, and reverse transcriptase controls prepared by substituting reverse transcriptase for nuclease-free water in the cDNA synthesis step were included in all analyses for each primer pair. A RT-qPCR checklist listing additional technical information as proposed by Bustin et al. [18], [19] is provided in Table S1.

Two publicly available software programs, geNorm and NormFinder were used to analyze Cq values which were collected from the ABI PRISM 7500 Sequence Detection System. Cq values were converted into relative quantities for geNorm and NormFinder taking into account the PCR efficiency of the primer pairs. These values were then used as software inputs and were analyzed as specified by Vandesompele et al [10] and Andersen et al [8].

### Normalization of the target gene

The oleosin gene (GenBank: AY962832), which encodes an oil body associated protein used as a stabilizer of oil bodies, was used for validating the impact of the use of inappropriate reference genes on the gene expression analysis. The primers were designed with Primer 5 software design (Forward: GCCACCAGGGGCTGACCAA and Reverse: CCTTCCCAGCCAACTTATGACG). The qPCR procedure was carried out following the same parameters used for the analysis of reference genes. The relative expression level of the target gene was calculated with different normalization factors in nine Siberian Apricot Germplasms employing the stable and unstable single gene and the reference genes recommended by geNorm and NormFinder program.

## Results

### Primer selection and amplification specificity and efficiency

Eleven Siberian Apricot reference genes commonly used as internal genes for plant gene expression studies were found via screening the Siberian Apricot EST database. Those genes were *ACT*, *TUA*, *TUB*, *CYP*, *DNAj*, *ELFA*, *UBQ*, *GAPDH*, ubiquitin-conjugating enzyme (*UBC*), ribosomal protein L12 (*RPL12*) and f-box protein 27 (*F-box27*) most of which represent different functional classes and gene families, with the possible exception of *TUA and TUB*; *UBC* and *UBQ*. Based on information about *Fragaria vesca subsp* genes, these potential internal genes intron/exon structures were then fabricated, drawing from other plant genome sequences. Then PCR primers were designed on different exons or spanning an exon-exon junction (Table 2).

**Table 2:** Description of Siberian Apricot candidate reference genes.

Gene^a^	Homologous species	Homologous Locus	% homology	E vuale	Primers location^b^
*ACT*	*Fragaria vesca subsp*	XM_004294460.1	90%	0	N/F
*TUA*	*Fragaria vesca subsp*	XM_004289661.1	91%	0	3/4
*TUB*	*Fragaria vesca subsp*	XM_004294187.1	88%	0	2/3
*CYP*	*Fragaria vesca subsp*	XM_004289796.1	87%	2e-174	N/F
*DNAj*	*Populus trichocarpa*	XM_002316443.1	86%	1e-121	2–3/4
*ELFA*	*Fragaria vesca subsp*	XM_004309835.1	92%	0	1–2/2
*GAPDH*	*Vitis vinifera*	XM_002263109.2	84%	0	9/10
*RPL12*	*Fragaria vesca subsp.*	XM_004291528.1	87%	6e-156	N/F
*UBC*	*Fragaria vesca subsp.*	XM_004287962.1	84%	5e-162	4/5
*UBQ*	*Fragaria vesca subsp*	XM_004294293.1	87%	0	N/F

a A*CT*, *TUA*, *TUB*, *CYP*, *DNAj*, *ELFA* and *UBQ* were ESTs based on the other species reference gene sequence determined via BLASTN.

b The prediction of exon, the previous digits indicated the site of primer forward and the back correspond to primer reverse. N/F meaning no intron or limited ESTs could not conjecture the intron.

**Figure 1:**
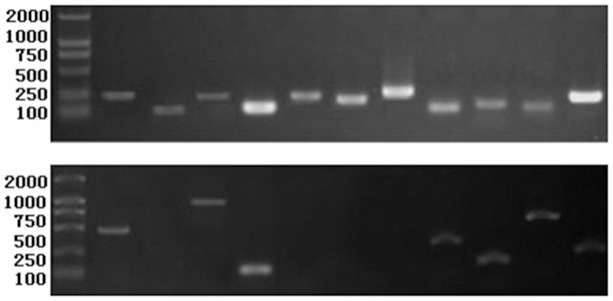
Performance of the amplification primers. Amplicons obtained by real-time PCR using cDNA (up) and gDNA (down) as template and electrophoresis using agarose gel (1.5%). The amplification primers from left to right are *ACT*, *TUA*, *TUB*, *CYP*, *DNAj*, *ELFA*, *F-box27*, *RPL12*, *GAPDH*, *UBC* and *UBQ*.

Agarose gel electrophoresis and melting curve analyses were performed following the RT-qPCR experiment, to determine specificity of primers designed in the current study. The primer pairs all amplified the expected size of the single PCR product (Figure 1). In addition, the specificity of amplicon was confirmed by the presence of a single peak during the melt curve and sequencing analysis (Figure S2). Average amplification efficiency varied from 99.2% and 105.9% (Table 3).

**Table 3:** Reference genes and their primer sequences used for real-time PCR.

Abbreviation	Gene name	Accession No.	Primer forward(F)/reverse(R)	Primer reverse	Amplicon size (bp)	E^b^ (%)	R^2^ b
*ACT*	Actin	CV049956.1	ACATTGTTCTTAGTGGTGGGTC	AGATTCGTCATACTCTGCCTTT	209	100.5±4.8	0.994
*TUA*	Alpha-tubulin	CV046479.1	TTGACATTGAGCGACCCACC	TCACATCCACATTCAGAGCACC	108	100.5±2.8	0.997
*TUB*	Beta-tubulin	CV045221.1	CTTGACAATGAAGCCCTCTATGA	AGTAAGAGGAGCAAAGCCCAC	219	104.6±5.7	0.995
*CYP*	Cyclophilin	CV046015.1	CAACGGATCTCAGTTCTTCGTCTGC	GACCCAACCTTCTCGATGTTCTTCA	120	101.8±2.1	0.998
*DNAj*	Chaperone protein dnaJ	CV052151.1	GGTGGACACGACCCATTTGA	ACCTGACTTTGACCCTTTACCC	219	105.1±4.7	0.998
*ELFA*	Elongation factor 1-alpha	CV046439.1	ACTGGAACCTCACAGGCTGAC	GGAGTAGTGGCATCCATCTTGTTA	170	105.9±3.6	0.993
*F-box 27*	F-box protein 27	EU836687.1	CGTGGAGTGATTTGATTGGC	AAGTTTGGGTGGTGGAGGC	106	99.8±5.5	0.997
*GAPDH*	Glyceraldehyde-3-phosphate	JN786944.1	ATGTCTTTCCGTGTTCCTACTGT	TTTCCCTCAGACTCCTCCTTG	116	100.4±3.5	0.993
*RPL12*	Ribosomal protein L12	U93168.1	CGATCCCTCACAGGTCGTCG	TCCAGTCGTTGGCGGTCTCC	149	99.2±3.3	0.990
*UBC*	Ubiquitin-conjugating enzyme	AF008910.1	GAGACCAGCAATAACCGTGAA	TCTTGTACTCCGTGGCATCCT	128	99.2±4.2	0.997
*UBQ*	Polyubiquitin	CB821710.1	CTCTGACTGGCAAGACCATAACA	CCACGGAGACGAAGGACAA	205	102.0±1.9	0.998

b PCR efficiency (E) and correlation coefficients (R^2^) were calculated by LinRegPCR method.

It was verified by PCR in both cDNA and gDNA whether these amplification primers were available. The primers for *TUA*, *DNAj*, *ELFA* and *F-box27* were unable to get amplified bands when using gDNA as template, and the amplifications of *ACT*, *TUB*, *GAPDH* and *UBC* genes with a gDNA template were longer than those obtained with a cDNA template. Thus those PCR primers were identified on different exons or spanning an exon-exon junction. In addition, the specific amplified bands of *CYP*, *RPL12* and *UBQ*, had the same size in cDNA and gDNA (Figure 1) as expected (Table 2). Although the experimental result of *ACT* gene deviated from our forecast in which the amplification of *ACT* should have had the same size, the remaining amplification primers on the list were exactly as we expected them to be.

### Expression levels of the reference genes

**Figure 2:**
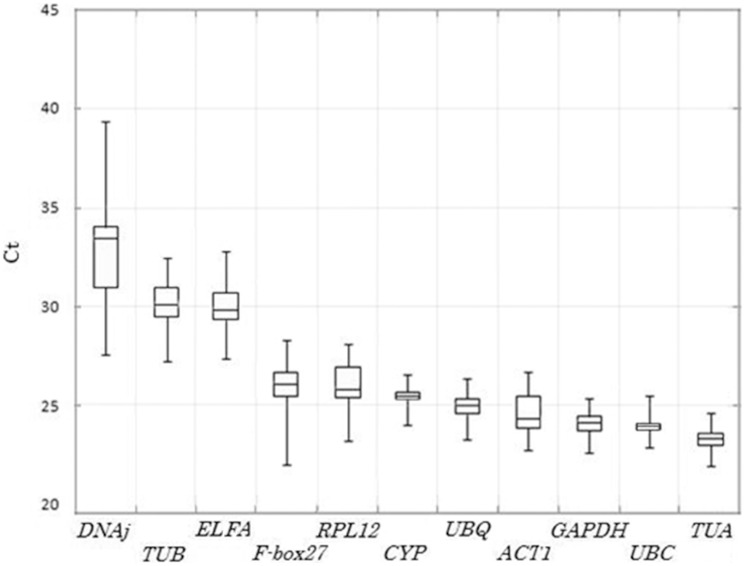
Cq values for 11 candidate reference genes across experimental samples. A line across the box is depicted as the median. The box indicates the 25th and 75th percentiles. Whiskers represent the maximum and minimum values.

To exclude any artificial errors in real-time PCR analysis of all the 11 candidate reference genes, three technical repetitions were performed for real-time PCR using gene-specific primers in the same cDNA pool. Meanwhile non-template controls were performed in parallel with each template and primer combination. The results showed that the single PCR product was amplified by each primer combination of the 11 candidate reference genes form various cDNA templates (Figure S2). Quantification cycle (Cq) values (the number of cycles needed for the fluorescence to reach a specific threshold level of detection) were determined in order to make comparison among each PCR run. The 11 candidate reference genes showed a relatively wide range of expression level from the lowest mean Cq value in *TUA* (23.36) to the highest in *DNAj* (32.56) with the most lying between 24 and 28 across all tested samples (Figure 2).

### Expression stability of reference genes

In order to identify and rank the most suitable reference genes based on their expression stability, the entire Cq dataset was analyzed using two different statistical approaches (geNorm and NormFinder).

**Figure 3:**
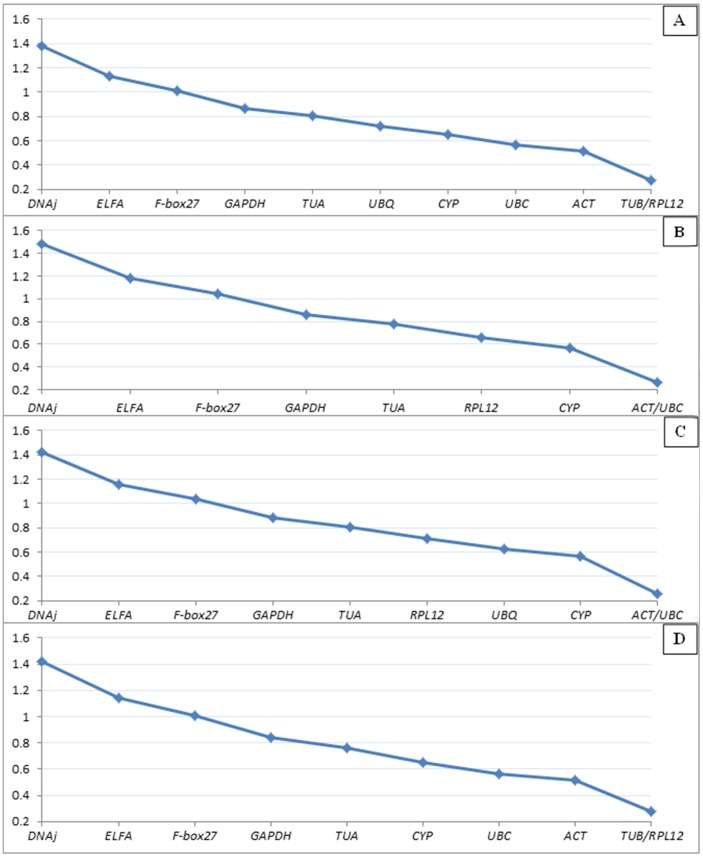
geNorm analysis of candidate reference genes in Siberian Apricot Germplasms. A: all reference genes. B: *TUB* and *UBQ* excluded from analysis. C: *TUB* excluded from analysis. D: *UBQ* excluded from analysis.

**Figure 4:**
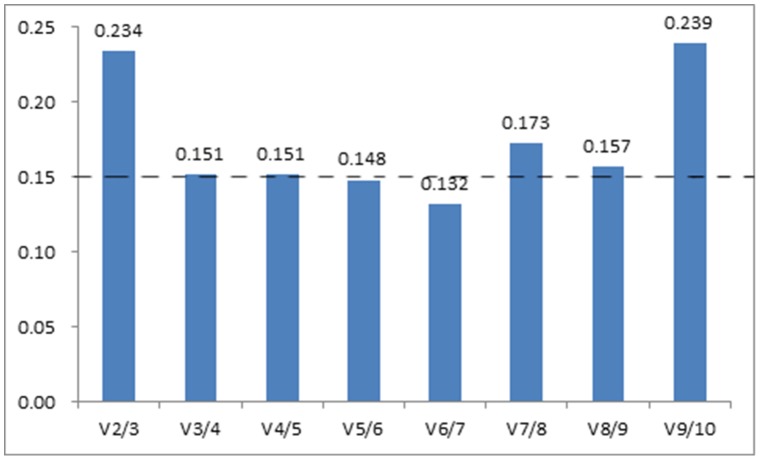
Pairwise variation analysis of normalization factors to determine the optimal number of reference genes. The cut-off value of 0.15, below which the inclusion of an additional reference gene is not required, is indicated by a discontinuous line.

The expression stability value of the 11 candidate reference genes was calculated by the geNorm program and then the reference genes *TUB* and *RPL12* were identified as the two most stably-expressed genes (Figure 3A), according to their M values defined as the average pairwise variation of that gene relative to all other potential reference genes in a set of cDNA samples. Since two pairs of reference genes (*TUB* and *TUA*; *UBQ* and *UBC*) were suspected to be coregulated, we removed both *TUB* and *UBQ* from analysis. The occurrence of a shift in ranking after the removal of those suspected coregulated genes (*TUB* and *UBQ*) showed that *ACT* and *UBC* were the best pair (Figure 3B). To determine whether the two pairs of reference genes were coregulated genes, *TUB* and *UBQ* were independently removed (Figure 3C and Figure 3D). The resulting ranking indicated that *TUB* and *TUA* were coregulated. In general, selecting more than two reference genes as an internal control can help significantly with the correction experimental error, providing get more reliable results. This is critical for accurate quantification of the genes, especially small expression differences in gene expression studies [20]. In order to select the ideal number of reference genes required for accurate normalization, the pairwise variation (Vn/Vn+1) was calculated by the geNorm algorithm. Vn values were calculated by stepwise inclusion of more reference genes until the (n+1) gene made no significant contribution to the newly calculated normalization factor [21]. According to the analysis, a value of 0.15 is usually regarded as the selection threshold value, for example V2/3<0.15 means that 2 reference genes could be used for normalization [10]. In our present study, V5/6 (0.148)<0.15<V4/5 (0.151) suggested that five reference genes were the best option for accurate normalization (Figure 4). However, we found the value of V3/4 (0.151) to be very close to 0.15, and three reference genes were used for attempted normalization. Finally, *UBC*, *ACT*, *CYP*, *UBQ* and *RPL12* were identified as the most suitable reference genes, and *UBC*, *CYP* and *ACT* were tentatively selected for normalization by geNorm.

**Figure 5:**
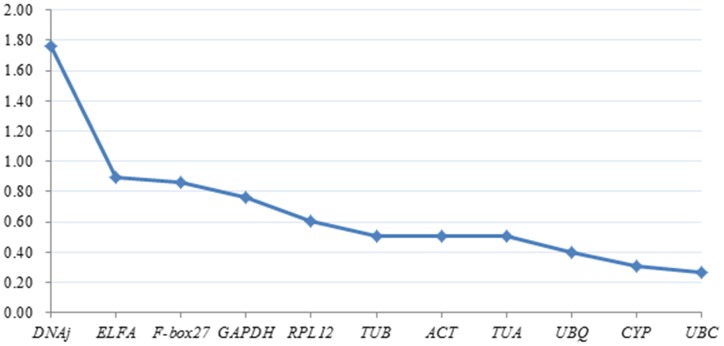
NormFinder analysis of candidate reference genes in Siberian Apricot Germplasms.

For a more robust analysis, we used the NormFinder approach, which is less affected by correlated expression compared with geNorm. A low stability value is indicative of stable expression; the NormFinder analysis provided roughly the same ranking of the candidate genes as geNorm without *TUB* (Figure 5). Based on our data, NormFinder identified the pair of *UBC* and CYP as the best one combination of reference genes in different Siberian Apricot Germplasms, and *F-box27, ELFA, DNAj* ranked consistently poorly.

### Expression profiling of oleosin

**Figure 6:**
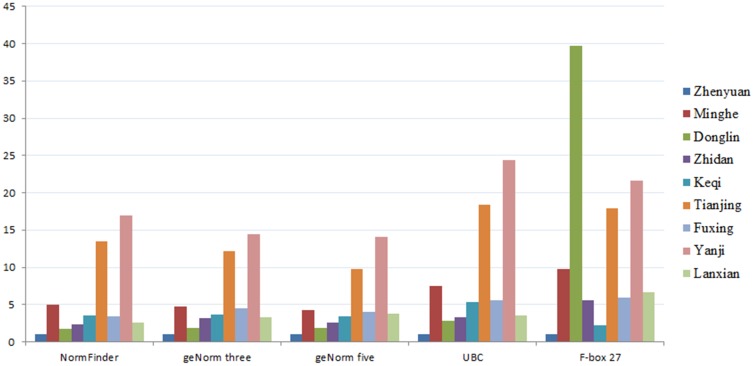
Expression profiles of oleosin gene in different Siberian Apricot Germplasms. Expression ratios of oleosin for the experimental calculated using (1) *CYP* and *UBC* for NormFinder; (2) *CYP*, *UBC* and *ACT* for geNorm; (3) *ACT*, *UBC*, *CYP*, *UBQ* and *RPL12* for geNorm; (4) the stable reference gene *UBC*; (5) the unsatisfactory reference gene *F-box27*.

The expression level of the oleosin gene was performed as an example to show the effect of the choice of reference genes on the expression profiling of other genes. The oleosin expression was analyzed in different Siberian Apricot Germplasms by using the various combinations of top reference genes indicated by each of the three methods: *UBC*, *CYP* and *ACT*, and *CYP*, *UBQ*, *ACT*, *RPL12* and *UBC* as indicated by geNorm; *CYP* and *UBC* as indicated by NormFinder. In addition, we also used the optimal (*UBC*) and the inadequate (*F-box27*) genes as reference genes. As shown in Figure 6, normalization using the unstable reference *F-box27* gene led to erroneous estimation of the target gene. When the *UBC* was used as a unique reference, the expressions of oleosin in Tianjing and Yanji were significantly overestimated. The results from geNorm (three and five genes) and NormFinder had a better parallel veracity.

## Discussion

The expression stability of the endogenous reference genes used as internal controls is paramount to reliable quantification of gene transcripts [22]. However, it has been reported that the reference genes are not only maintaining basic cellular functions but also participate in other cellular functions, generally resulting in theirs expressions considerable variation under different experimental conditions [23]. Therefore, it is necessary to validate the expression stability of potential reference genes in each particular experimental background prior to their use for normalization.

Siberian Apricot, belonging to the family Rosaceae, is also an important fruit and oilseed tree in China. Fast growth, easy propagation, high yield, and a high percentage of unsaturated fatty acids and a wide variety of bioactive components in the kernels make this plant one of the major resource of the formulation of protective lotion and soaps [13], [14]. Also, owing to its kernel oil associated with a reduced risk of chronic diseases [24], Siberian Apricot is considered to be one of the most intensively studied tree species. However, most of the researches for Siberian Apricot kernel oil and bioactive components rest only on the level of detection [25], [26]. Until now, little is known about the genetic and molecular level duo to a lack of information on reference gene stability in a variety of experimental contexts, limiting the application of RT-qPCR in the Siberian Apricot. Though 26S rRNA gene was used as a reference gene for normalization of real-time PCR data in examining carotenoid biosynthesis of apricots [15], systematic exploration and validation of stable Siberian Apricot reference genes had never been performed. In addition, the ribosomal RNA genes will not be able to participate as reference genes where cDNA synthesis is carried out using an oligo-dT primer or where only mRNA is used as template. Furthermore, they may exhibit such high levels of expression which would result in the occurrence of experimental error when normalizing weakly expressed genes [27]. All the pitfalls described above led us to not include the ribosomal RNA genes among the candidate reference genes to be evaluated in the present study.

In this present study, we used geNorm and NormFinder algorithms to select and validate the best reference genes form 11 candidates in Siberian Apricot Germplasms (including much variability) by calculating the expression stability of reference genes and determining the number of reference genes required for accurate normalization across the experimental conditions tested. It well known that geNorm and NormFinder relies on many of the same principles for expression variability evaluation across a panel of candidate reference genes, but NormFinder is less affected by correlated expression of the candidate genes. In contrast, one major defect in geNorm is insensitive to coregulated reference genes, leading the candidate reference genes to be preferentially selected from different pathways and functional classes [10]. However, it is sometimes difficult to avoid using coregulated genes for geNorm, especially when dealing with poorly annotated genes, or genes of unknown or hypothetical function [8]. Actually, the coregulated genes, such as *TUB* and *TUA*, were found in our experiments (Figure 3), as the case of previous studies [8], [28], suggesting that error analysis must be applied to ensure reliable results when using a set of multiple reference genes that unintentionally involve coregulated genes. Therefore, when coregulated genes are absent, geNorm and NormFinder normally provide the same general ranking, with only minor differences in ordering. This was proven in our results (Figure 3C and Figure 5) in accordance with previous reports [29]. We also verified that the combination of two or more reference genes improves the stability value compared to the single most stable gene (Figure 6), *ACT*, *UBC*, *CYP*, *UBQ* and *RPL12* were ultimately identified by geNorm, *UBC* and *CYP* by NormFinder. Interestingly, *UBC* and *CYP* were ranked as the most stable by geNorm and NormFinder. In fact *UBC* was also the optimal for all developmental stages and under all stress conditions in *Platycladus orientalis* [30], but it showed less stable expression in rice grown under various environmental conditions [31]. As noted previously, *CYP* was the worst reference gene in a diverse pool of poplar [32], however the optimal stability of *CYP* expression identified in our study was in agreement with the result of *Petunia hybrid* transcriptomic analysis [33]. Additionally, the observations on the reference genes *ACT*, *UBQ* and *RPL2* with stable expression in peach (Rosaceae) [34], *Brassica oleracea* [35] and tomato [28], respectively, had been completely verified in our studies. It has been reported that *ELFA* was the most stable in potato during biotic and abiotic stress [36], but poorly ranked during light stress in tomato [28]. *ELFA* was top ranked for the developmental stage series and different times of the day, but poorly ranked for different tissues under the same developmental stage in soybean [37]. In our analysis results, *ELFA* performed poorly by geNorm and NormFinder enlightened us that *ELFA* was the most variable gene. Moreover, according to the other two genes (*DNAj* and *F-box27*) with poor rank validated in our current experimental conditions, it can be concluded that three genes (*DNAj*, *F-box27* and *ELFA*) should not be used as reference genes in Siberian Apricot Germplasms.

To evaluate the effect of the choice of reference genes generated by two statistical methods on the expression profiling of genes this study, the four kinds of reference genes, including the optimal reference gene (*UBC*), the unsatisfactory one (*F-box27*), and the combination (*ACT*, *UBC*, *CYP*, *UBQ* and *RPL12*) identified by geNorm as well as another one (*UBC* and *CYP*) by NormFinder, were used to analyze the relative expression of the oleosin gene in, encoding the most abundant protein in oil bodies of plants [38]. As illustrated in Figure 6, although he kinds and amounts of reference genes differently picked by the geNorm algorithm (a set of several genes) and NormFinder (only one or two), the oleosin expression levels quantified with references selected according to either geNorm or NormFinder were conformance, as reported by Løvdal and Saha's report [29]. This was probably due to the less affected by correlated expression for NormFinder, which imply that NormFinder may be the preferred choice for stability evaluation if the experimenter is unsure about coregulation among a set of candidate references [29]. Additionally, an attempt was made to single out three reference genes (*ACT*, *CYP* and *UBC*) from candidate references by geNorm in this study, mainly due to the value of V3/4 (0.151) very close to 0.15, from which we were surprised to find that the three reference genes also could obtain satisfactory results. Thus, from a view of the economy and accuracy, it is concluded that a set of three genes (*ACT*, *CYP* and *UBC*) could be used as the best control for normalization in Siberian Apricot Germplasms.

## Conclusions

As far as can be ascertained, this is the first systematic study for the selection of reference genes for RT-qPCR in Siberian Apricot Germplasms. The evaluations of 11 candidate reference genes by geNorm and NormFinder indicated that the three most suitable reference genes in Siberian Apricot Germplasms are *ACT*, *UBC* and *CYP*, while the three least suitable reference genes are *DNAj*, *ELFA* and F-box27. To obtain the most reliable results from gene expression studies of Siberian Apricot, the combination of three or more stable genes should be used as internal controls for relative gene quantification.

## Supporting Information

Figure S1 **The distribution of nine Siberian Apricot Germplasms.**
(TIF)

Figure S2 **Dissociation curves for candidate reference genes along with NTC.**
(TIF)

Table S1 **The information of MIQE.**
(XLS)
