# Supplementary figures and images for: Selection of Reference Genes for Gene Expression Studies in Siberian Apricot (Prunus sibirica L.) Germplasm Using Quantitative Real-Time PCR
Source: PLoS One. 2014 Aug 8;9(8):e103900. doi: 10.1371/journal.pone.0103900 (PMC4126684; doi:10.1371/journal.pone.0103900)

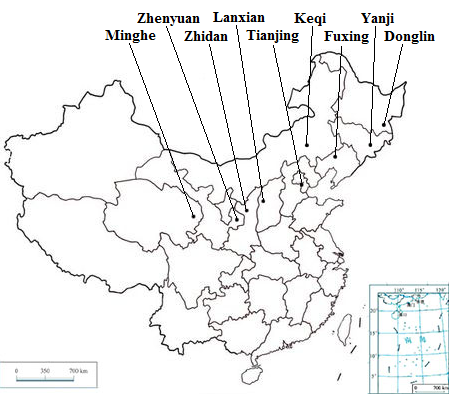

Supplement: Figure S1 — The distribution of nine Siberian Apricot Germplasms. (TIF) [file pone.0103900.s001.tif]

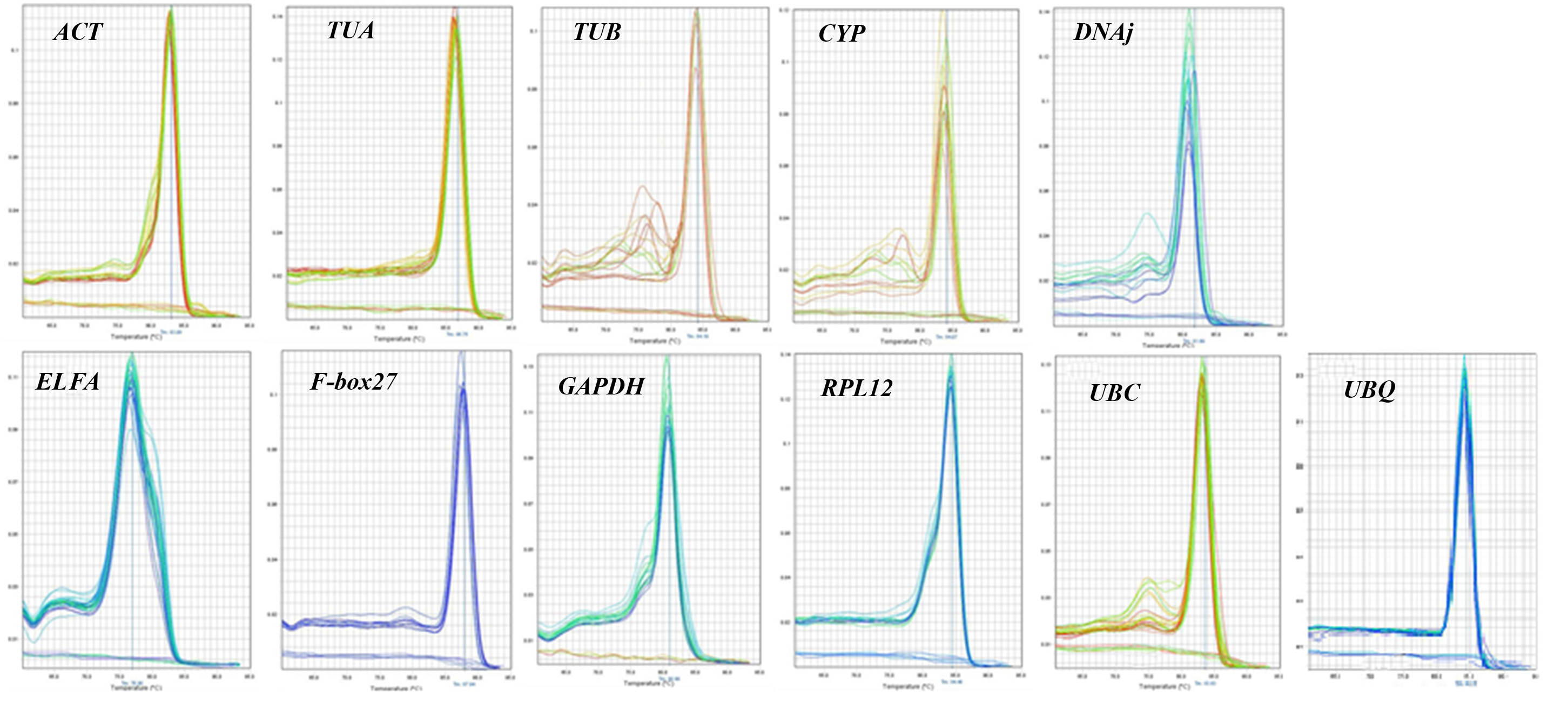

Supplement: Figure S2 — Dissociation curves for candidate reference genes along with NTC. (TIF) [file pone.0103900.s002.tif]
